# Inequities in Stroke Recovery: Examining Sociodemographic Predictors of Rehabilitation Success

**DOI:** 10.3390/healthcare13141739

**Published:** 2025-07-18

**Authors:** Suzana Dedijer Dujović, Olivera Djordjević, Aleksandra Vidaković, Sindi Mitrović, Mirko Grajić, Tijana Dimkić Tomić, Stefan Rosić, Ana Radić, Ljubica Konstantinović

**Affiliations:** 1 Clinic for Rehabilitation “Dr. Miroslav Zotovic”, Sokobanjska 13, 11000 Belgrade, Serbia; aleksandra.dragin@med.bg.ac.rs (A.V.); sindi.mitrovic@med.bg.ac.rs (S.M.); tijana.dimkic-tomic@med.bg.ac.rs (T.D.T.); stefan.rosic.86@gmail.com (S.R.); ana.radic@rehabilitacija.rs (A.R.); ljubica.konstantinovic@med.bg.ac.rs (L.K.); 2 Faculty of Medicine, University of Belgrade, 11000 Belgrade, Serbia; grajicm@gmail.com; 3 Center for Physical Medicine and Rehabilitation, University Clinical Center of Serbia, 11000 Belgrade, Serbia

**Keywords:** sociodemographic factors, stroke, rehabilitation, functional recovery

## Abstract

**Background:** Stroke recovery is influenced not only by clinical but also sociodemographic factors (SDFs). However, data on how variables such as age, sex, marital status, education, and employment status affect rehabilitation outcomes remain limited, particularly in structured inpatient settings. This study aimed to analyze the impact of key SDFs on functional recovery after stroke. **Methods:** A retrospective cohort of 289 stroke patients undergoing structured inpatient rehabilitation was analyzed. Functional status was assessed at admission, after three weeks, and at discharge using five standardized outcomes: gait speed (primary outcome), Barthel Index, Berg Balance Scale, Action Research Arm Test, and Ashworth scale. Repeated measures ANOVA and multivariable logistic regression were used to evaluate within-subject changes and associations with SDFs. **Results:** The cohort consisted predominantly of middle-aged to older adults (58% female, 62% married, 60% retired, 60% with primary education or less). Most patients (88%) had ischemic strokes of moderate severity. Significant improvements were observed across all functional measures. Employed, married, younger, and male patients achieved better outcomes. Interaction models indicated that older and female patients with moderate stroke severity demonstrated greater improvement than younger and male counterparts with milder strokes. Mean gait speed increased by +0.32 m/s, exceeding the minimal clinically important difference (MCID) of 0.16 m/s. **Conclusions:** Age, sex, marital status, education, and employment status are relevant predictors of stroke rehabilitation outcomes. These findings emphasize the importance of incorporating sociodemographic profiles into individualized rehabilitation planning to optimize functional recovery and reduce disparities among stroke survivors.

## 1. Introduction

Stroke remains a leading cause of adult disability and functional dependence worldwide, posing an increasing global burden, particularly in low- and middle-income countries [1]. Functional recovery after stroke significantly depends on timely, intensive, and multidisciplinary rehabilitation interventions designed to restore mobility, self-care abilities, and participation in everyday life [2]. However, recovery trajectories vary widely among individuals, influenced not only by clinical parameters—such as lesion size, stroke type, and initial neurological deficit—but also by a variety of non-clinical determinants, notably sociodemographic factors [3].

Sociodemographic status (SDS) is a multidimensional construct encompassing educational attainment, occupational position, income level, living conditions, residential area, and access to healthcare services, including health insurance coverage [4]. Contemporary evidence consistently indicates that individuals from lower SDS backgrounds encounter significant barriers in accessing specialized stroke rehabilitation services [5]. These barriers may manifest as delays in receiving appropriate therapeutic interventions, limited availability of specialized rehabilitation facilities, and consequently prolonged functional recovery times [6,7]. Furthermore, lower educational levels, often associated with reduced health literacy, can impede patients’ understanding of therapeutic recommendations and negatively affect adherence to prescribed rehabilitation regimens, thus amplifying disparities in stroke recovery outcomes [8].

Although research increasingly supports associations between SDS and stroke outcomes, the precise mechanisms and the specific SDS dimensions that are the most influential on functional recovery remain unclear. Furthermore, there is a notable lack of studies simultaneously assessing multiple SDS domains and their interactions with objective functional measures—such as gait speed, balance, upper limb function, and independence in activities of daily living—particularly in structured inpatient rehabilitation settings.

Given the global socioeconomic shifts, especially pronounced in low- and middle-income regions, understanding how these sociodemographic factors influence stroke recovery is essential for developing targeted and equitable health policies aimed at ensuring equal access to rehabilitation and minimizing outcome disparities. This study aims to explore the influence of key sociodemographic factors—sex, age, marital status, education level, and employment status—on functional recovery in stroke patients undergoing structured inpatient rehabilitation.

## 2. Materials and Methods

### 2.1. Study Design and Setting

This retrospective cohort study included patients with a diagnosis of stroke who were hospitalized at the Clinic for Rehabilitation “Dr Miroslav Zotović” in Belgrade between January 2017 and December 2019. The study sample comprised 289 patients with a confirmed first-ever stroke diagnosis, verified through neuroimaging (CT or MRI). Exclusion criteria encompassed patients with comorbidities that could significantly interfere with rehabilitation outcomes. These included severe cognitive impairment (e.g., dementia), hemodynamic instability, uncontrolled cardiac arrhythmias, musculoskeletal injuries, and pre-existing neurological or systemic diseases associated with impaired motor function. The study was approved by the institutional ethics committee, and all patient data were anonymized prior to analysis

### 2.2. Data Collection

For this study, the following variables were analyzed: Baseline confounders were sex female/male) and patient age at the time of stroke. Data included type of stroke, listed as either ischemic (as defined by the Oxford Community Stroke Project [9]) or hemorrhagic; admission stroke severity according to the National Institutes of Health Stroke Scale (NIHSS); location of stroke; and side of hemiparesis or hemiplegia. The National Institutes of Health Stroke Scale (NIHSS) was collected on hospital discharge to identify stroke severity after acute stroke treatment. An NIHSS score of 0 point was considered a no-stroke symptom, 1 to 4 points a minor stroke, 5 to 15 points a moderate stroke, 16 to 20 points a severe stroke, and 21 to 42 points a very severe stroke [10].

Sociodemographic status (SDS) was assessed through a multidimensional approach incorporating five key variables: age, sex, marital status, educational attainment, and employment status. The selected SDFs were chosen based on their well-established predictive value for stroke outcomes, their frequent examination in the stroke rehabilitation literature, and their feasibility for routine clinical assessment [5,6]. Age was stratified into four clinically relevant categories, under 55 years, 55–65 years, 65–75 years, and over 75 years, to account for variations in recovery potential across the lifespan. Sex was recorded as a binary variable (female/male). Marital status was categorized as married or single, with the latter group including unmarried, divorced, or widowed individuals. Educational attainment was dichotomized into two levels, elementary education (up to 8 years of formal schooling) and secondary education or higher (9 or more years of schooling), reflecting potential differences in health literacy and engagement with rehabilitation protocols. Employment status at the time of stroke was classified as employed, unemployed, or retired, to capture differences in occupational activity, social participation, and potential economic support. These SDS indicators were included as independent variables in the statistical analysis to examine their associations with functional recovery trajectories

To evaluate the functional status of patients, several standardized assessment tools were utilized at three distinct time points: upon admission, after three weeks of rehabilitation, and at discharge. The primary outcome of this study was gait speed, selected for its strong predictive value for independence and its sensitivity to changes during rehabilitation, as well as its strong association with social determinants [11]. Gait speed was assessed over a 10 m distance. To eliminate the influence of acceleration and deceleration, patients walked along a 12 m walkway, with the first and last meter excluded from the timed segment. Secondary functional outcomes included the Barthel Index [12], Berg Balance Scale [13], Action Research Arm Test (ARAT) for upper limb function [14], and the Ashworth scale for muscle tone assessment [15].

### 2.3. Data Analyses

All statistical analyses were conducted using R Studio software (Version 1.4.1106; RStudio, PBC, Boston, MA, USA, 2009–2021). Both descriptive and inferential statistics were applied to summarize and evaluate the data. Categorical variables were analyzed using the chi-square (χ^2^) test or Fisher’s exact test, depending on expected frequencies.

To assess changes in functional outcome scores across three time points (pre-test, retest, post-test), one-way repeated measures ANOVA was employed. Post hoc pairwise comparisons were conducted using the Bonferroni correction to control for multiple testing.

Two-way repeated measures ANOVA was applied to explore potential interaction effects between sociodemographic status variables and time on functional outcomes. A significant interaction implies that the effect of one factor (e.g., SDS variable) on the outcome depends on the level of the other factor (e.g., time), and vice versa. For categorical repeated measures data, Cochran’s Q test was used. To examine the association between SDS factors and the probability of functional improvement, multivariable binary logistic regression analysis was performed. Interaction terms were included in the regression models where relevant.

All statistical tests were two-tailed, and a *p*-value less than 0.05 was considered statistically significant.

## 3. Results

### 3.1. General Characteristics of Participants

**Figure 1:**
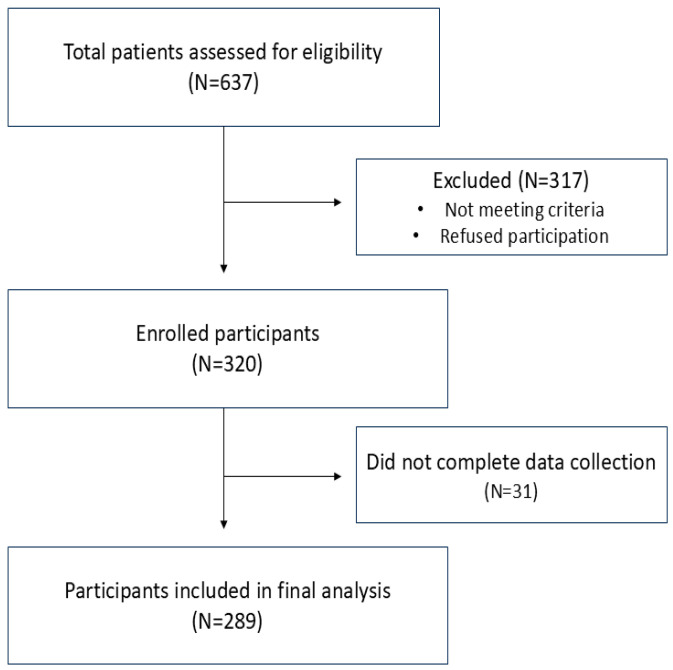
Flow diagram of the study protocol.

This study included a total of 289 post-stroke patients, 58% of whom were male and 42% female. Most participants were aged between 55 and 75 years (64%), with 30% aged 55–65 and 34% aged 65–75. The majority of respondents were retired (60%), while 29% were employed and 11% unemployed. Regarding marital status, 62% were married and 38% single. Most participants had primary or lower education (60%), while 40% had secondary or higher education. Overall, 8% of subjects had no education level, 52% had elementary school education, 19% subjects finished high school, 17% had college-level education, and only 4% were faculty. Figure 1 shows a flow diagram of patient recruitment and retention through the study. Descriptive statistics of sociodemographic status are presented in Table 1.

Descriptive statistics of lateralization, localization, and stroke type are presented in Table 2. More than half the subjects, 54%, had right-side motor deficit, while 42% had left-side. Only 11% had lateralization on both sides. Most participants had subcortical stroke localization (45%), followed by cortical (24%), bilateral (18%), cerebellar (8%), and lacunar (5%) involvement. Most of the participants (88%) had ischemic while 12% had hemorrhagic stroke.

### 3.2. Influence of Socioeconomic Factors on Functional Recovery

#### 3.2.1. Gait Speed (Speed)

As the primary outcome, gait speed showed statistically significant improvement across all three assessment points (pre, retest and post) F (333.5,1.3) = 245.7; *p* < 0.0001. Post hoc analysis with a Bonferroni correction was performed to assess pairwise differences between time points, all showing *p* < 0.0001 (Table 3).

A comprehensive repeated measures analysis of gait speed across different sociodemographic (SD) categories revealed statistically significant changes over time for all examined groups (*p* < 0.0001) (Table 4). Employed participants showed significantly higher gait speeds than retirees at retest (*p* = 0.03) and post-test (*p* = 0.007). Male participants had significantly higher values than females at all time points, with significant differences at retest (*p* = 0.003) and post-test (*p* = 0.004). Married participants had higher gait speeds than single individuals at all time points, with the greatest differences at post-test (*p* = 0.00008). Regarding age, participants younger than 55 years had the highest gait speeds, while those older than 75 had the lowest. Statistically significant differences between age groups were observed at retest (*p* = 0.004) and post-test (*p* = 0.013).

Two-way repeated measures ANOVA indicated statistically significant interaction effects between time and sociodemographic status (F (333.7, 1.3) = 3.6, *p* = 0.04), sex (F (334, 1.3) = 4.8, *p* = 0.02), marital status (F (334, 1.3) = 5.4, *p* = 0.01), and age (F (364.4, 3.8) = 2.8, *p* = 0.02). These interactions suggest that the pattern of change in gait speed over time differed across SD groups.

Multivariable logistic regression showed that both the NIHSS score and several sociodemographic factors were significantly associated with gait speed improvement: NIHSS score (β = −0.25; OR = 0.78, 95% CI: 0.71–0.89, *p* = 0.04), female sex (β = −0.64; OR = 0.56, 95% CI: 0.31–0.89, *p* = 0.01), and marital status (married: β = 0.48; OR = 1.61, 95% CI: 0.95–2.76, *p* = 0.04) (Table 5).

According to the negative coefficient for NIHSS (β = −0.25) with the odds ratio 0.78, we may assume that the probability of developing improvements in speed for subjects with a higher NIHSS score is 22% smaller than for those with a lower NIHSS score. The negative beta coefficient for female sex (β = −0.64) with OR = 0.56 means that the probability of developing an improvement in gait speed for the female subjects is 47% smaller than for the male subjects. The positive beta coefficient for the married subjects in our sample (β = 0.48) with OR = 1.61 means that their probability of developing an improvement in speed is 61% higher than for the single subjects.

The multivariable logistic regression model with interaction terms revealed that patients with moderate stroke (NIHSS) aged over 75 had a 4.09-fold higher chance of improvement compared to younger individuals with mild stroke (*p* = 0.04). Women with moderate stroke had a 2.64-fold higher chance of improvement than men with mild stroke (*p* = 0.04), while married patients with moderate stroke were 4.79 times more likely to improve than single individuals with mild stroke (*p* = 0.02).

#### 3.2.2. Barthel Index

The Barthel Index score was statistically significantly different at the three points (pre, retest, and post) F (367.6; 1.3) = 290.5; *p* < 0.0001. Post hoc analysis with a Bonferroni correction was performed the pairwise differences between time points with *p* < 0.0001 (Table 3).

Analysis of variance (ANOVA) and independent sample *t*-tests revealed statistically significant differences in the Barthel Index between employed and retired participants at retest (*p* = 0.03) and post-test (*p* = 0.014), with employed individuals showing significantly better scores (Table 6). Statistically significant differences between men and women were observed only at post-test (*p* = 0.03). Married participants had significantly higher scores at retest (*p* = 0.03), while no significant differences were observed at pre-test and post-test. Differences across age groups were significant at all three time points: pre-test (*p* = 0.0001), retest (*p* = 0.0003), and post-test (*p* = 0.001). Two-way repeated measures ANOVA indicated a statistically significant interaction between age and time (F (364.4, 3.8) = 2.8; *p* = 0.02). No significant interactions were observed for sex (F (365, 1.3) = 0.64; *p* = 0.46), marital status (F (366, 1.3) = 0.31; *p* = 0.63), or employment status (F (366, 1.3) = 0.09; *p* = 0.82) (Table 6).

Multivariable logistic regression showed no statistically significant association between any of the examined sociodemographic characteristics and the Barthel Index score (*p* > 0.05 for all variables).

#### 3.2.3. Berg Balance Scale

One-way repeated measures ANOVA showed statistically significant differences in Berg Balance Scale scores during rehabilitation F (339.5; 1.3) = 300.3, *p* < 0.0001. Post hoc analysis with Bonferroni correction was performed to assess the pairwise differences between time points with *p* < 0.0001 (Table 3).

Analysis of variance (ANOVA) and independent sample *t*-tests revealed statistically significant differences in Berg Balance Scale scores between employed and retired participants at all three time points, pre-test (*p* = 0.0004), retest (*p* = 0.0006), and post-test (*p* = 0.0002), with employed participants achieving higher scores (Table 7). Statistically significant differences were also observed between men and women at pre-test (*p* = 0.001), retest (*p* = 0.0002), and post-test (*p* = 0.00003), with male participants having higher scores. Married participants scored significantly higher than single participants at retest (*p* = 0.047) and post-test (*p* = 0.013), while no significant difference was observed at pre-test. Significant differences were found across all age categories at each time point (pre-test, retest, and post-test; all *p* < 0.0001).

Two-way repeated measures ANOVA indicated a statistically significant interaction between employment status and time (F (338, 1.3) = 0.5; *p* = 0.04). No statistically significant interactions were found for sex (F (339, 1.3) = 0.08; *p* = 0.83), marital status (F (339, 1.3) = 1.6; *p* = 0.22), or age (F (336, 3.8) = 0.5; *p* = 0.73) (Table 7).

Multivariable logistic regression showed no statistically significant independent association between SD factors and improvement on the Berg Scale. The logistic regression model with interaction terms showed that married patients with moderate stroke had a 3.25-fold higher chance of improvement on the Berg Scale compared to single patients with mild stroke (*p* = 0.03).

#### 3.2.4. ARAT Scale

One-way repeated measures ANOVA revealed a statistically significant improvement in upper limb function, measured by ARAT scores, across the three assessment time points (*p* < 0.0001) (Table 3).

Two-way repeated measures ANOVA showed a statistically significant interaction effect between employment status and time (F (323, 1.2) = 4.6; *p* = 0.02), indicating that the trajectory of upper limb recovery differed between employed and retired participants. However, no statistically significant interaction was found between sex and time (F (322, 1.2) = 0.5; *p* = 0.52), nor between marital status and time (F (323, 1.2) = 3.2; *p* = 0.06). Age also did not demonstrate a significant interaction with time (F (321, 3.7) = 1.9; *p* = 0.11). Similarly, education level did not show a statistically significant interaction with time (F (322, 1.2) = 1.56; *p* = 0.21) (Table 8).

Multivariable logistic regression did not reveal any significant associations between SD variables and improvement on the ARAT scale.

#### 3.2.5. Ashworth Scale (Muscle Tone)

Analysis of spasticity using the modified Ashworth scale demonstrated a statistically significant reduction in muscle tone during the course of inpatient rehabilitation. One-way repeated measures ANOVA confirmed this improvement for both upper and lower limbs (*p* < 0.0001). Mean Ashworth scores decreased progressively from admission to discharge, indicating a clinically meaningful reduction in spasticity. Detailed values are presented in Table 9. However, two-way repeated measures ANOVA revealed no statistically significant interactions between sociodemographic factors and time, suggesting that the observed changes in muscle tone were consistent across different sociodemographic subgroups.

A reduction in lower limb spasticity was more likely among employed and married patients, suggesting the role of social engagement and support in modulating post-stroke muscle tone. Other sociodemographic factors showed no significant effect in this domain (Table 10).

Analysis of the Ashworth scale for upper limbs showed no statistically significant influence of any SD factor.

### 3.3. Correlation Between NIHSS Score and Functional Outcomes by SD Variable

Significant negative correlations were consistently observed between initial NIHSS scores and all functional outcomes (Barthel Index, gait speed, Berg Balance Scale, and ARAT), with the strongest correlations generally found among employed and middle-aged participants. Specifically, employed participants exhibited the strongest correlations across all outcomes (Barthel r = −0.58; Speed r = −0.52; Berg r = −0.49; ARAT r = −0.54; all *p* < 0.001) (Table S1).

Moderate correlations were noted among pensioners, while the unemployed group showed strong correlations except for ARAT, which was not significant. Regarding age, correlations were strongest in the 55–75-year age range, notably with the Barthel Index (55–65 years, r = −0.63) and gait speed (65–75 years, r = −0.62), while no significant correlations were found in the group aged over 75 (Table S1).

Sex, marital status, and education level also influenced these relationships. Women had slightly stronger correlations with gait speed (r = −0.46) and Berg Balance scores (r = −0.43) compared to men, whereas men had stronger correlations with ARAT scores (r = −0.35). Married participants generally showed stronger correlations than single participants with gait speed and Berg Balance scores, but single participants had stronger ARAT correlations. Higher education was associated with stronger gait speed correlations, while participants with elementary education showed notably stronger correlations for the Berg Balance Scale (Table S1).

## 4. Discussion

The results of this study emphasize the significance of sociodemographic determinants in shaping functional recovery after stroke, particularly age, sex, marital status, educational attainment, and employment status. Younger participants demonstrated consistently better outcomes across all domains, likely reflecting greater neuroplasticity, fewer comorbidities, and improved physiological resilience. Male patients achieved superior improvements in walking speed and balance, supporting the need for sex-sensitive rehabilitation approaches that address both biological and social disparities. Marital status also emerged as a relevant factor, with married individuals showing better recovery—likely due to continuous emotional and practical support from partners, which enhances motivation and reduces social isolation. Although educational attainment did not consistently predict functional outcomes in our sample, possibly due to the structured nature of inpatient rehabilitation diminishing differences in health literacy, a trend toward better recovery among those with higher education levels was noted. Employment status showed the strongest association, as employed individuals had significantly better outcomes across multiple domains, likely reflecting stronger baseline health, greater motivation, and a clear goal of returning to work. Among all outcome measures, gait speed proved to be particularly sensitive to sociodemographic variation, showing consistent associations with age, sex, marital status, and employment status, thereby reinforcing its value as a clinically meaningful indicator of mobility and independence [11]. Similarly, though variably strong, associations were also observed for the Barthel Index, Berg Balance Scale, ARAT, and Ashworth scores.

Age has long been recognized as one of the most influential predictors of stroke recovery, with numerous studies reporting that younger patients tend to achieve more favorable outcomes. This advantage is usually attributed to enhanced neuroplasticity, preserved motor function, and a lower burden of comorbidities, all of which increase responsiveness to therapy [16,17]. In contrast, older adults often face frailty, cognitive decline, and reduced physical endurance, factors that can delay or attenuate functional gains [18]. They are also less likely to be referred to intensive programs, and when they are, they frequently receive fewer therapy hours or less aggressive treatment [19]. Beyond these biological considerations, age interacts with other sociodemographic variables that can further shape recovery. Older patients are disproportionately retired, which may limit daily structure, financial resources, and social networks, thereby dampening engagement in rehabilitation [20,21]. Marital status also moderates age effects: widowhood and social isolation are more common in later life and can undermine emotional well-being and motivation [22,23]. Educational attainment—often lower in older cohorts because of historical trends—may reduce health literacy and adherence to exercise prescriptions, further influencing trajectories [3,8]. Consistent with this multifactorial picture, our younger participants scored higher across all functional domains—gait speed, balance, upper limb dexterity, and activities of daily living—while older groups progressed more slowly. Because we did not age-standardize our analyses, residual confounding between age and other sociodemographic factors may persist. Future studies should apply age-standardized models or incorporate interaction terms to disentangle these effects and to refine age-adapted, socially sensitive rehabilitation pathways that can mitigate disparities and optimize outcomes for older stroke survivors [24].

Sex-based disparities in stroke recovery have long been documented in the literature, with growing recognition that biological, behavioral, and social factors may contribute to differential outcomes between women and men. Several large-cohort studies have reported that women often experience poorer post-stroke functional recovery, potentially due to older age at stroke onset, greater pre-stroke disability, and higher prevalence of comorbidities such as hypertension or atrial fibrillation [25,26]. Additionally, psychosocial determinants, including differences in social support, caregiver availability, and access to rehabilitation services, may further influence gender-related recovery patterns [27,28]. In our study, men consistently achieved better outcomes than women across functional domains, particularly in gait speed and balance scores. These results are in line with previous findings suggesting that men may benefit from higher baseline physical function and greater engagement in rehabilitation activities [29]. Taken together, these observations underscore the importance of developing sex-sensitive rehabilitation strategies that consider both physiological and social determinants of recovery.

Marital status has consistently been identified as a relevant psychosocial determinant of post-stroke recovery. Numerous studies have shown that individuals who are married or cohabiting tend to achieve better functional outcomes following a stroke, which is often attributed to the increased availability of emotional support, practical assistance, and encouragement to adhere to rehabilitation regimens [22]. Spousal support has been associated with improved motivation, reduced risk of post-stroke depression, and more consistent engagement in therapy sessions, all of which contribute to more favorable recovery trajectories [30]. Our study found that married participants demonstrated superior functional recovery, particularly in gait speed, balance, and independence measures. These results corroborate existing evidence linking marital status to better stroke outcomes, potentially mediated by reduced social isolation—a known risk factor for poorer recovery. The findings underscore the need to integrate family-centered approaches into rehabilitation protocols, extending beyond caregiver involvement to actively strengthen patients’ social support networks [31,32], which are associated with significantly better psychological and functional outcomes [23]. Clinically, this highlights the pivotal role of familial support in optimizing recovery and suggests that addressing social dynamics should be a core component of post-stroke care.

Educational attainment is often cited as a key predictor of stroke recovery, largely due to its association with health literacy, cognitive reserve, and self-management capacity. Patients with higher education levels may be more likely to understand therapeutic instructions, adhere to rehabilitation regimens, and engage more actively in recovery, all of which contribute to better outcomes [8]. Moreover, higher education has been linked to improved access to digital health tools and more proactive health behaviors that support long-term rehabilitation [4]. The World Stroke Organization has also highlighted education as a structural determinant of health inequality and a driver of stroke burden and recovery outcomes globally [1]. In our study, however, educational level did not consistently predict functional outcomes across the measured domains. This finding contrasts with previous research—such as a large Chinese cohort study demonstrating that low education level independently predicted higher mortality and stroke recurrence (hazard ratio 2.8 for all-cause mortality in individuals with illiteracy vs. college education) and may be explained by several contextual factors [33]. First, the structured nature of inpatient rehabilitation may mitigate the impact of individual differences in health literacy by offering standardized therapy regardless of background. Second, a large proportion of our cohort had only primary education, limiting between-group variability and potentially underpowering any true effect. Third, educational attainment may act through related sociodemographic factors—such as employment status and age—which showed stronger and more consistent associations in our models. These findings suggest that while education is an important theoretical determinant of recovery, its observable effect may vary depending on the setting and population.

Employment status has been increasingly recognized as an important sociodemographic factor influencing post-stroke recovery. Previous studies indicate that employed individuals often experience better rehabilitation outcomes compared to their unemployed or retired counterparts due to several potential mechanisms, such as greater financial stability, structured daily activities, stronger social networks, and enhanced psychological well-being [20,34]. Stable employment post-stroke provides critical structure and purpose, significantly enhancing motivation and therapy adherence. Swedish studies demonstrate that 45–56% of patients return to work, with employment status strongly correlating to rehabilitation engagement [21]. Our findings strongly align with these prior observations, demonstrating significantly superior functional outcomes among employed participants in multiple assessments, notably gait speed and balance performance.

In addition to the observed associations between sociodemographic factors and outcomes assessed by the Barthel Index, gait speed, Berg Balance Scale, and ARAT, our findings emphasize the predictive strength of the NIHSS score as a clinical determinant of functional recovery. Muscle tone, evaluated using the Ashworth scale for both upper and lower limbs, also demonstrated significant reduction during rehabilitation, reflecting meaningful clinical improvement, although without statistically significant interactions with sociodemographic variables. Functional outcomes such as gait speed and overall independence were significantly correlated with both SES variables and stroke severity, suggesting a complex interaction between clinical and social factors in shaping recovery trajectories. NIHSS scores exhibited significant negative correlations with all functional outcomes across multiple SDS strata, particularly among employed participants and middle-aged groups, confirming its role as a core prognostic indicator in stroke rehabilitation. These findings are consistent with prior studies where higher NIHSS values are associated with worse functional outcomes [3]. Moreover, interaction models revealed nuanced effects. Patients aged over 75 with moderate stroke severity had a 4.09-fold higher chance of improvement in gait speed compared to younger patients with milder strokes. Similarly, women with moderate strokes had a 2.64-fold greater probability of progress compared to men with milder deficits, while married individuals with moderate strokes showed a 4.79-fold increased likelihood of functional improvement relative to their single counterparts. These results suggest that stroke severity does not operate in isolation, but that its impact is modified by demographic and social contexts, warranting tailored rehabilitation strategies that consider both biological and socioeconomic profiles.

The choice of gait speed as the primary outcome measure was based on its clinical relevance, sensitivity to change, and strong predictive value for long-term function and independence [11,35]. Its established association with multiple sociodemographic determinants further supported its use as a meaningful recovery indicator. However, the selection of a measure so closely linked to sociodemographic characteristics may have amplified observed associations and introduced interpretative bias. It should also be acknowledged that pre-stroke physical activity levels and environmental barriers—both of which vary by socioeconomic context—may partially mediate the relationship between sociodemographic factors and gait speed outcomes [34,35]. Future research should incorporate multiple complementary outcome measures and include data on premorbid physical activity and environmental context in order to reduce potential bias and clarify these complex mediating pathways. The observed mean improvement in gait speed from baseline to discharge was + 0.32 m/s, which exceeds the established minimal clinically important difference (MCID) of 0.16 m/s for post-stroke populations. This indicates that the functional gains observed were not only statistically significant, but also clinically meaningful and likely to translate into real-world improvements in mobility and independence [36,37].

This study has several limitations that should be acknowledged. First, mood variables such as depression and anxiety, which are known to affect rehabilitation outcomes and may correlate with sociodemographic factors, were not assessed [23,30]. Their exclusion limits the ability to distinguish between emotional and structural influences on recovery. Second, although gait speed was chosen as the primary outcome due to its strong clinical validity and sensitivity to change, its established association with sociodemographic factors may have introduced interpretative bias. Additionally, unmeasured factors such as pre-stroke physical activity levels and environmental barriers could have mediated the observed relationships [34,35]. Third, our analyses did not apply age standardization, which may have resulted in residual confounding when interpreting the interactions between age and other sociodemographic variables. Future studies should address these limitations by integrating psychological measures, capturing environmental and lifestyle data, and applying standardized modeling techniques.

It is also essential to acknowledge that our study exclusively analyzed patients who successfully accessed structured inpatient rehabilitation. Consequently, the sample may not fully reflect the broader population of stroke survivors, particularly those unable to access rehabilitation services due to socioeconomic or geographic constraints. Previous research has identified marked disparities in healthcare access across Serbia, especially among rural and economically disadvantaged populations, which may have influenced both baseline characteristics and recovery potential at admission [38]. This limitation highlights the need for future research focused on identifying and addressing access barriers in stroke rehabilitation services.

## 5. Conclusions

In conclusion, our study highlights the essential role of sociodemographic factors in shaping stroke rehabilitation outcomes. Recognizing key determinants such as age, sex, marital status, employment status, and educational attainment enables the development of personalized rehabilitation strategies that address patient-specific needs and reduce health disparities. These insights support a shift toward more socioeconomically informed rehabilitation planning. Future studies should focus on elucidating the mechanisms underlying these associations and on validating targeted interventions designed to improve recovery outcomes across diverse sociodemographic groups of stroke survivors.

## Figures and Tables

**Table 1:** Descriptive statistics of sociodemographic status of subjects.

Variable	Categories	N (%)	Subcategories	N (%)	
Age	Below 55 y	52 (18%)			
	55–65 y	88 (30%)			
	65–75 y	98 (34%)			
	Above 75 y	51 (18%)			
Sex	Female	122 (42%)			
	Male	167 (58%)			
Marital Status	Married	180 (62%)	Married		180 (62%)
	Single	109 (38%)	Unmarried		56 (20%)
			Widow		40 (14%)
			Divorced		13 (4%)
Education	Elementary	174 (60%)	Without elementary e.		23 (8%)
			Elementary e.		151 (52%)
	High	115 (40%)	High school		55 (19%)
			College		49 (17%)
			Faculty		11 (4%)
Working Status	Employed	115 (40%)	Employed		83 (29%)
			Unemployed		32 (11%)
	Pensioner	174 (60%)	Pensioner		174 (60%)

**Table 2:** Descriptive statistics of stroke lateralization, lesion sites, and etiological classification of subjects.

Variable	Categories	n (%)
Lateralization	Right	157 (54%)
	Left	121 (42%)
	Both side	11 (4%)
Localization	Cortical	69 (24%)
	Subcortical	131 (45%)
	Bilateral	52 (18%)
	Lacunae	13 (5%)
	Cerebellum	24 (8%)
Stroke type	Ischemic	254 (88%)
	Hemorrhagic	35 (12%)

**Table 3:** Changes in functional outcome scores across three time points.

Outcome	Time	η_1_ ± sd_1_	η_2_ ± sd_2_	Test Statistics *	*p* Value *	F Value ^#^	*p* Value ^#^
Speed	Pre ^1^–Retest ^2^	0.39 ± 0.36	0.55 ± 0.38	−13.6	<0.0001	F (333.5, 1.3) = 245.7	<0.0001
	Pre ^1^–Post ^2^	0.39 ± 0.36	0.71 ± 0.44	−14.2	<0.0001		
	Retest ^1^–Post ^2^	0.55 ± 0.38	0.71 ± 0.44	−16.9	<0.0001		
BI	Pre ^1^–Retest ^2^	58.4 ± 31.4	70.1 ± 27	−15.6	<0.0001	F (367.6, 1.3) = 290.5	<0.0001
	Pre ^1^–Post ^2^	58.4 ± 31.4	80.2 ± 24	−18.2	<0.0001		
	Retest ^1^–Post ^2^	70.1 ± 27	80.2 ± 24	−14.9	<0.0001		
BBS	Pre ^1^–Retest ^2^	29.3 ± 17.4	37.1 ± 15.6	−15.5	<0.0001	F (339.5, 1.3) = 300.3	<0.0001
	Pre ^1^–Post ^2^	29.3 ± 17.4	42.8 ± 14.7	−18.2	<0.0001		
	Retest ^1^–Post ^2^	37.1 ± 15.6	42.8 ± 14.7	−15.9	<0.0001		
ARAT	Pre ^1^–Retest ^2^	36.5 ± 23.7	40.6 ± 22.2	−7.65	<0.0001	F (323.5, 1.3) = 323.5	<0.0001
	Pre ^1^–Post ^2^	36.5 ± 23.7	43.1 ± 21.7	−8.7	<0.0001		
	Retest ^1^–Post ^2^	40.6 ± 22.2	43.1 ± 21.7	−6.95	<0.0001		

η_1_ ± sd_1_—mean and standard deviation of 1; η_2_ ± sd_2_—mean and standard deviation of 2; *—test statistics and *p* value of multicomparison tests; ^#^—test statistics and *p* value of the one-way repeated ANOVA; speed—gait speed m/s; BI-Barthel Index; BBS-Berg Balance Scale; ARAT-Action Research Arm Test.

**Table 4:** The effect of sociodemographic status variables and time on gait speed.

SDF	Categories	Time			Multicomparison			Interaction
		Pre *	Retest *	Post *	Pre-Retest ^#^	Pre-Post ^#^	Retest—Post ^#^	SDvar: Time
Age	Below 55 y	0.42 ± 0.38	0.65 ± 0.38	0.87 ± 0.45	<0.0001	<0.0001	<0.0001	F (364.4, 3.8) = 2.8 *p* = 0.02
	55–65 y	0.45 ± 0.4	0.58 ± 0.42	0.74 ± 0.46	<0.0001	<0.0001	<0.0001	
	65–75 y	0.38 ± 0.33	0.52 ± 0.36	0.66 ± 0.42	<0.0001	<0.0001	<0.0001	
	Above 75 y	0.29 ± 0.32	0.46 ± 0.32	0.61 ± 0.4	<0.0001	<0.0001	<0.0001	
	Comparison ^#^	0.152	0.09	0.01				
Sex	Female	0.33 ± 0.33	0.47 ± 0.35	0.6 ± 0.41	<0.0001	<0.0001	<0.0001	F (334, 1.3) = 4.8 *p* = 0.02
	Male	0.44 ± 0.37	0.61 ± 0.38	0.8 ± 0.44	<0.0001	<0.0001	<0.0001	
	Comparison ^#^	0.02	0.003	0.004				
Marital status	Married	0.44 ± 0.39	0.62 ± 0.4	0.8 ± 0.43	<0.0001	<0.0001	<0.0001	F (334, 1.3) = 5.4 *p* = 0.01
	Single	0.32 ± 0.3	0.45 ± 0.33	0.52 ± 0.42	<0.0001	<0.0001	<0.0001	
	Comparison ^#^	0.008	0.0006	0.00008				
Education	Elementary	0.4 ± 0.37	0.55 ± 0.38	0.71 ± 0.43	<0.0001	<0.0001	<0.0001	F (332, 1.3) = 0.13 *p* = 0.78
	High	0.38 ± 0.34	0.55 ± 0.38	0.72 ± 0.45	<0.0001	<0.0001	<0.0001	
	Comparison ^#^	0.78	0.99	0.99				
Working status	Pensioner	0.36 ± 0.33	0.51 ± 0.34	0.66 ± 0.4	<0.0001	<0.0001	<0.0001	F (333.7, 1.3) = 3.6; *p* = 0.04
	Employment relationship	0.44 ± 0.4	0.62 ± 0.43	0.81 ± 0.47	<0.0001	<0.0001	<0.0001	
	Comparison ^#^	0.122	0.03	0.007				

* mean ± standard deviation; ^#^—*p* value.

**Table 5:** The multivariable logistic regressions—gait speed.

Coefficients	Estimate	Std. Error	Z Value	*p* Value
Age	−0.02	0.01	−1.16	0.25
Sex	−0.64	0.27	−2.39	0.01
Marital	0.48	0.27	1.94	0.04
Education	−0.26	0.26	−0.97	0.33
Working	−0.05	0.35	−0.13	0.89
NIHSS	−0.25	0.14	−1.93	0.04
Intercept	0.95	0.79	1.19	0.23

*p* value of Cochran’s Q tests; test statistics of Cochran’s Q tests.

**Table 6:** The effect of sociodemographic status variables and time on Barthel Index.

SDF	Categories	Time			Multicomparison			Interaction
		Pre *	Retest *	Post *	Pre-Retest ^#^	Pre-Post ^#^	Retest—Post ^#^	SDvar: Time
Age	Below 55 y	62.6 ± 27.8	73.8 ± 20.8	86.5 ± 16.2	<0.0001	<0.0001	<0.0001	F (364.4, 3.8) = 2.8 *p* = 0.02
	55–65 y	65.6 ± 29.8	76.7 ± 23.3	84 ± 19.4	<0.0001	<0.0001	<0.0001	
	65–75 y	58.3 ± 31.3	68.9 ± 28.7	78.6 ± 26.8	<0.0001	<0.0001	<0.0001	
	Above 75 y	41.8 ± 32.8	57.2 ± 31	70.1 ± 28.9	<0.0001	<0.0001	<0.0001	
	Comparison ^#^	0.0001	0.0003	0.001				
Sex	Female	56 ± 33.4	66.7 ± 29.3	76.7 ± 26.4	<0.0001	<0.0001	<0.0001	F (365, 1.3) = 0.64 *p* = 0.46
	Male	60.1 ± 29.9	72.6 ± 25	82.7 ± 22.1	<0.0001	<0.0001	<0.0001	
	Comparison ^#^	0.274	0.06	0.03				
Marital status	Married	60.8 ± 31.3	72.7 ± 26.9	82.2 ± 23.7	<0.0001	<0.0001	<0.0001	F (366, 1.3) = 0.31 *p* = 0.63
	Single	54.4 ± 31.4	65.8 ± 26.8	76.7 ± 24.2	<0.0001	<0.0001	<0.0001	
	Comparison ^#^	0.09	0.03	0.06				
Education	Elementary	56.8 ± 33.6	68.7 ± 29.1	78.8 ± 25.7	<0.0001	<0.0001	<0.0001	F (366, 1.3) = 0.02 *p* = 0.92
	High	60.7 ± 27.9	72.2 ± 23.5	82.3 ± 21.1	<0.0001	<0.0001	<0.0001	
	Comparison ^#^	0.314	0.115	0.226				
Working status	Pensioner	55.3 ± 32.2	67.3 ± 29	77.3 ± 26.4	<0.0001	<0.0001	<0.0001	F (366, 1.3) = 0.09 *p* = 0.82
	Employed	63 ± 29.8	74.3 ± 23.2	84.4 ± 19.3	<0.0001	<0.0001	<0.0001	
	Comparison ^#^	0.059	0.03	0.014				

* mean ± standard deviation; ^#^—*p* value.

**Table 7:** The effect of sociodemographic status variables and time on Berg Balance Scale.

SDF	Categories	Time			Multicomparison			Interaction
		Pre *	Retest *	Post *	Pre-Retest ^#^	Pre-Post ^#^	Retest—Post ^#^	SDvar: Time
Age	Below 55 y	37.6 ± 15.1	45.2 ± 10.6	50.7 ± 17	<0.0001	<0.0001	<0.0001	F (336, 3.8) = 0.5 *p* = 0.73
	55–65 y	31.5 ± 17	39.3 ± 14	45.7 ± 11.7	<0.0001	<0.0001	<0.0001	
	65–75y	28.3 ± 17.1	35.4 ± 16.1	41 ± 16.2	<0.0001	<0.0001	<0.0001	
	Above 75 y	20.2 ± 16.4	29.3 ± 17	34.3 ± 16.6	<0.0001	<0.0001	<0.0001	
	Comparison ^#^	<0.0001	<0.0001	<0.0001				
Sex	Female	25.4 ± 17.8	33.1 ± 16.9	38.6 ± 16.3	<0.0001	<0.0001	<0.0001	F (339, 1.3) = 0.08 *p* = 0.83
	Male	32.3 ± 16.5	40.2 ± 13.7	46 ± 12.5	<0.0001	<0.0001	<0.0001	
	Comparison ^#^	0.001	0.0002	0.00003				
Marital status	Married	30.3 ± 17.9	38.6 ± 15.6	44.5 ± 14.3	<0.0001	<0.0001	<0.0001	F (339, 1.3) = 1.6 *p* = 0.22
	Single	27.7 ± 16.5	34.7 ± 15.3	40 ± 15	<0.0001	<0.0001	<0.0001	
	Comparison ^#^	0.23	0.047	0.013				
Education	Elementary	27.8 ± 18.2	35.6 ± 16.8	41.6 ± 15.9	<0.0001	<0.0001	<0.0001	F (338, 1.3) = 0.32 *p* = 0.62
	High	31.6 ± 16	39.3 ± 13.3	44.6 ± 12.4	<0.0001	<0.0001	<0.0001	
	Comparison ^#^	0.08	0.06	0.09				
Working status	Pensioner	26.4 ± 17.2	34.5 ± 15.9	40.3 ± 15.7	<0.0001	<0.0001	<0.0001	F (338, 1.3) = 0.5; *p* = 0.04
	Employed	34 ± 16.8	41.2 ± 14.2	46.9 ± 11.9	<0.0001	<0.0001	<0.0001	
	Comparison ^#^	0.0004	0.0006	0.0002				

* mean ± standard deviation; ^#^—*p* value.

**Table 8:** The effect of sociodemographic status variables and time on ARAT.

SDF	Categories	Time			Multicomparison			Interaction
		Pre *	Retest *	Post *	Pre-Retest ^#^	Pre-Post ^#^	Retest—Post ^#^	SDvar: Time
Age	Below 55 y	36.9 ± 22.5	42 ± 20	46 ± 19.3	<0.0001	<0.0001	<0.0001	F (321, 3.7) = 1.9 *p* = 0.11
	55–65 y	34 ± 25.1	39.4 ± 23.6	42.4 ± 23.4	<0.0001	<0.0001	<0.0001	
	65–75 y	36.2 ± 23.4	39.2 ± 23.7	40.7 ± 23.3	<0.0001	<0.0001	<0.0001	
	Above 75 y	40.6 ± 21.6	44.1 ± 19.3	46.3 ± 17.8	<0.0001	<0.0001	<0.0001	
	Comparison ^#^	0.49	0.56	0.38				
Sex	Female	36.5 ± 24.6	40.5 ± 22.8	42.5 ± 22.5	<0.0001	<0.0001	<0.0001	F (339, 1.3) = 0.08 *p* = 0.83
	Male	36.5 ± 23	40.7 ± 21.7	43.6 ± 21.1	<0.0001	<0.0001	<0.0001	
	Comparison ^#^	0.99	0.95	0.68				
Marital status	Married	35.4 ± 24	40.3 ± 22.1	43.1 ± 21.8	<0.0001	<0.0001	<0.0001	F (323, 1.2) = 3.2 *p* = 0.06
	Single	35.6 ± 23	41.3 ± 22.3	43.2 ± 21.7	<0.0001	<0.0001	<0.0001	
	Comparison ^#^	0.31	0.72	0.96				
Education	Elementary	36.4 ± 23.7	40.1 ± 22.6	42.3 ± 22	<0.0001	<0.0001	<0.0001	F (322, 1.2) = 1.56 *p* = 0.21
	High	36.6 ± 23.7	41.5 ± 21.5	44.7 ± 21.2	<0.0001	<0.0001	<0.0001	
	Comparison ^#^	0.95	0.64	0.41				
Working status	Pensioner	37.5 ± 23.4	41 ± 22.2	42.8 ± 21.8	<0.0001	<0.0001	<0.0001	F (323, 1.2) = 4.6; *p* = 0.02
	Employed	34.7 ± 24.1	39.9 ± 22.2	43.7 ± 21.7	<0.0001	<0.0001	<0.0001	
	Comparison ^#^	0.36	0.7	0.76				

* mean ± standard deviation; ^#^—*p* value.

**Table 9:** Changes in Ashworth scores for muscle tone for upper and lower limb across three time points.

Ashworth Arm	Time				
Categories **	Pre	Retest	Post	*p* Value *	χ^2^
4	1 (1%)	1 (1%)	1 (1%)	<0.0001	35.9
3	5 (2%)	3 (1%)	1 (1%)		
2	14 (5%)	11 (4%)	11 (4%)		
1+	21 (8%)	20 (8%)	17 (6%)		
1	77 (29%)	83 (32%)	72 (28%)		
0	144 (55%)	144 (55%)	160 (61%)		
3	1 (1%)	1 (1%)	2 (1%)	<0.0001	19
2	10 (4%)	5 (2%)	9 (4%)		
1+	21 (8%)	18 (7%)	14 (5%)		
1	92 (34%)	95 (35%)	85 (31%)		
0	147 (54%)	152 (55%)	161 (59%)		

* *p* value of Cochran’s Q tests; test statistics of Cochran’s Q tests; ** 0—no increase in tone; 1—slight increase in tone giving a catch when the limb is moved in flexion or extension; 1+—slight increase in muscle tone, indicated by a catch followed by minimal resistance throughout range of motion (ROM); 2—more marked increase in tone through most of the ROM, but the limb easily flexes; 3—considerable increase in tone, and passive movement difficult; 4—limb rigid in flexion or extension.

**Table 10:** The multivariable logistic regressions—the Ashworth lower limb.

Coefficients	Estimate	Std. Error	Z Value	*p* Value
Age	0.03	0.02	1.53	0.13
Sex	−0.71	0.43	−1.66	0.09
Marital	−0.76	0.39	−1.97	0.04
Education	−1.15	0.39	−0.38	0.71
Working	−0.78	0.49	−1.98	0.04
Intercept	−2.74	1.2	−2.34	0.02

## Data Availability

The original contributions presented in this study are included in the article. Further inquiries can be directed to the corresponding author.

## Supplementary Materials

The following supporting information can be downloaded at: https://www.mdpi.com/article/10.3390/healthcare13141739/s1, Table S1. NIHSS Correlations with Functional Outcomes by SD Variables.

## Abbreviations

The following abbreviations are used in this manuscript:

SDS	Sociodemographic status
NIHSS	National Institutes of Health Stroke Scale
BI	Barthel Index
BBS	Berg Balance Scale
ARAT	Action Research Arm Test
