# Supplementary material for: Inequities in Stroke Recovery: Examining Sociodemographic Predictors of Rehabilitation Success
Source: Healthcare (Basel). 2025 Jul 18;13(14):1739. doi: 10.3390/healthcare13141739 (PMC12294742; doi:10.3390/healthcare13141739)
Supplement: Supplementary file 1 [file healthcare-13-01739-s001.zip › healthcare-3735674-supplementary.pdf]

Table S1. NIHSS Correlations with Functional Outcomes by SD Variables

	Between vars	category	p value	Corr coef
Age	Barthel post - NIHSS	Less 55y	0.004	-0.39
		55-65y	<0.001	-0.63
		65-75y	<0.001	-0.39
		Above 75y	0.12	-0.21
	Speed post – NIHSS	Less 55y	0.007	-0.4
		55-65y	<0.001	-0.47
		65-75y	<0.001	-0.62
		Above 75y	0.12	-0.23
	Berg – NIHSS	Less 55y	0.027	-0.33
		55-65y	<0.001	-0.45
		65-75y	<0.001	-0.51
		Above 75y	0.11	-0.23
Sex	Barthel post - NIHSS	Female	<0.001	-0.37
		Male	<0.001	-0.39
	Speed post – NIHSS	Female	<0.001	-0.46
		Male	<0.001	-0.40
	Berg – NIHSS	Female	<0.001	-0.37
		Male	<0.001	-0.43
	ARAT – NIHSS	Female	<0.001	-0.35
		Male	0.002	-0.28
Marital status	Barthel post - NIHSS	Married	<0.001	-0.39
		Unmarried	<0.001	-0.35
	Speed post – NIHSS	Married	<0.001	-0.48
		Unmarried	<0.001	-0.32
	Berg – NIHSS	Married	<0.001	-0.47
		Unmarried	<0.001	-0.29
	ARAT – NIHSS	Married	<0.001	-0.28
		Unmarried	<0.001	-0.39
Education	Barthel post - NIHSS	High e.	<0.001	-0.39
		Elementary e.	<0.001	-0.35
	Speed post – NIHSS	High e.	<0.001	-0.48
		Elementary e.	0.001	-0.32
	Berg – NIHSS	High e.	0.011	-0.25
		Elementary e.	<0.001	-0.48
	ARAT – NIHSS	High e.	0.007	-0.28
		Elementary e.	<0.001	-0.33

Working status	Barthel post - NIHSS	Employed	<0.001	-0.58
		Unemployed	0.037	-0.37
		Pensioner	<0.001	-0.43
	Speed post – NIHSS	Employed	<0.001	-0.52
		Unemployed	0.001	-0.58
		Pensioner	<0.001	-0.36
	Berg – NIHSS	Employed	<0.001	-0.49
		Unemployed	<0.001	-0.58
		Pensioner	<0.001	-0.43
	ARAT – NIHSS	Employed	<0.001	-0.54
		Unemployed	0.91	-0.03
		Pensioner	<0.001	-0.42

Speed- gait speed measured in meters per second (m/s), BI- Barthel Index, BBS- Berg Balance Scale, NIHSS- National Institutes of Health Stroke Scale, and ARAT Action Research Arm Test scored in points
